# Roadblocks confronting widespread dissemination and deployment of Organs on Chips

**DOI:** 10.1038/s41467-024-48864-3

**Published:** 2024-06-15

**Authors:** Charles G. Alver, Emma Drabbe, Matthew Ishahak, Ashutosh Agarwal

**Affiliations:** 1 Department of Biomedical Engineering, University of Miami, Coral Gables, FL USA; 2 Medical Scientist Training Program, University of Miami Miller School of Medicine, Miami, FL USA; 3 Desai Sethi Urology Institute, University of Miami Miller School of Medicine, Miami, FL USA

**Keywords:** Lab-on-a-chip, Biosensors, Microfluidics, Biomedical engineering

## Abstract

Organ on Chip platforms hold significant promise as alternatives to animal models or traditional cell cultures, both of which poorly recapitulate human pathophysiology and human level responses. Within the last 15 years, we have witnessed seminal scientific developments from academic laboratories, a flurry of startups and investments, and a genuine interest from pharmaceutical industry as well as regulatory authorities to translate these platforms. This Perspective identifies several fundamental design and process features that may act as roadblocks that prevent widespread dissemination and deployment of these systems, and provides a roadmap to help position this technology in mainstream drug discovery.

## Abstract (web summary)

Organ on Chip platforms hold significant promise as alternatives to traditional animal models or cell cultures. In this Perspective, the authors examine the barriers that prevent widespread dissemination and deployment of these systems.

## Introduction

While traditional 2D culture methods have served the scientific community well over the past century, the development of Organ on Chip devices has improved the ability to culture more complex 3D engineered and ex-vivo tissues^1–6^. Conceptually, Organ on Chip devices are continuously perfused microfluidic devices used to culture cells or engineered tissues in a way that models tissue- and organ-level functions within a minimal functional unit. These microfluidic devices can range from simple systems with one or more related cell types^7–10^ to more complex multichannel systems that utilize porous membranes and multiple cell cultures to mimic tissue interfaces, such as those in the lungs or kidney^11–15^. Multiple Organ on Chip devices have been linked together to analyze the interactions between different organ functions in the context of drug or toxin delivery as well as metabolism and other multiorgan processes^5,16–21^. For example, vasculature or immune cells can be co-cultured with various types of organoids^22^. More complex models can be created by fusing different types of organoids generated separately to reconstitute the interaction of multiple tissues^23,24^. A notable example of a complex Organ on Chip is the EVATAR system for female reproductive systems^14,25–27^, which includes separate organ modules for the ovary, fallopian tube, uterus, cervix, and liver within a microfluidic system to simulate the in vivo female reproductive tract. The development of new Organ on Chip devices centers around the incorporation of physiologically relevant mechanical and fluidic parameters, such as shear stress, mechanical strain and compression, and perfusate composition, which includes but is not limited to additional suspended cells (e.g. immune cells), stimulants (e.g. hormones and drugs), and gaseous species (e.g. oxygen and carbon dioxide)^1,5,28^. The inclusion of flow has been shown to help mature cell cultures by mimicking the phenotypical characteristics of the comparable in-vivo system^1,29^. Additionally, Organ on Chip devices are designed to utilize an ever-growing array of analytic techniques to assess the functionality and biomechanics of the tissues cultured within them, such as live-cell imaging, analyte collection, and electrophysiological readouts^2,3,16,29–32^. The continued advancement of microfabrication and tissue engineering technologies has helped to make Organ on Chip devices an optimizable cell culture platform capable of recapitulating the in-vivo micro-physiological environment of a variety of organ systems.

With more robust recapitulation of physiological microenvironments compared to traditional 2D cultures, Organ on Chip devices have begun playing a more significant role in translational science work, especially within the field of drug development. The minimal functional unit of an organ that exists within an Organ on Chip provides an accurate physiological microenvironment that is essential for drug development and risk profiling^29,33^. Recapitulation of a specific disease microenvironment ensures that the efficacy of a drug—both in terms of its function and its targeting to a specific organ system—can be appropriately validated in the early stages of drug development^34–36^. When compared to animal models commonly used at this early stage of drug development, Organ on Chip models can be more effective at identifying potentially useful novel therapeutics because these models use tissues and cell lines derived from human samples^33,37^. A human-based drug discovery model ensures that when potential new drugs are identified, the drug actually targets the human receptor or pathology. Without this validation of human specificity, prior drug discovery trials have fallen flat once they move past animal testing into pre-clinical testing^38^. Additionally, these human Organ on Chip models provide a significantly more accurate analysis of drug toxicities for potential new drugs compared to animal models in the pre-clinical phase, since they are also able to demonstrate side effects in human tissue that might not be present in animal models^36,38–40^. Aside from the biological benefits to drug discovery, Organ on Chip devices can also be run at a high throughput, meaning that many potential new drugs can be tested in rapid succession or even concurrently^41–43^. Because of these physiological and experimental advantages of Organ on Chip devices over traditional animal models, Organ on Chip devices offer a promising new pathway within the pharmaceutical industry.

## Outline

Traditional animal models and cell cultures both poorly recapitulate human pathophysiology and human level responses. Organ on Chip platforms, on the other hand, allow researchers to exert control over tissue composition and architecture by providing an array of cellular and extracellular cues to then precisely recapitulate the minimal functional unit of human tissues or organs. This decade has witnessed seminal scientific developments from academic laboratories, a flurry of startups that seek to translate those platforms, and a genuine interest from pharmaceutical industry as well as regulatory authorities. The individual and collaborative efforts of these important stakeholders will undoubtedly continue to refine and mature Organ on Chip platforms. It is imperative, therefore, to identify any fundamental design and process features that might ultimately prevent widespread dissemination and deployment of these systems. In this perspective, we examine several such barriers. First, we discuss the various stakeholders in the development of Organs on Chips, and their roles and responsibilities throughout the process. Second, we highlight the complications associated with polydimethylsiloxane (PDMS), the widely used material for Organ on Chip fabrication and discuss alternatives. Third, we discuss the engineering design process that will facilitate fully functional Organ on Chip platforms. Finally, we examine the pathway to adoption by the pharmaceutical industry, especially given the FDA Modernization Act 2.0. We submit that recognition and rectification of these barriers is especially timely, as these technologies are beginning to be tested and validated by the intended end-users: disease biologists, pharmaceutical companies, and regulatory authorities.

## The translational landscape of Organ on Chip technology

Following the landmark publication of Lung on a Chip in 2010, and robust support from funding agencies, Organ on Chip technology has become one of the fastest growing areas in bioengineering^12^. While the foundational use of Organs on Chips can be attributed to the academic convergence of microfluidics, stem cell technology and tissue engineering^44,45^, one of the major current driving forces is the interest of pharmaceutical companies in applying this technology to drug development. As a result, an ecosystem of new biotechnology companies has emerged to help move Organs on Chips out of academic research labs and into the drug discovery space^33,46^. In the US, startup companies have been spun out of academic institutions primarily focused on providing research services that utilize unique Organ on Chip models^47^. In Europe, the expansion of Organ on Chip technology is being driven by 3D organoid companies and microfluidic companies looking to add Organ on Chip applications to their portfolios^48^. The value proposition for many of these startups focuses on providing better data for lead optimization^49^. However, Organ on Chip platforms face many technical and regulatory barriers that may complicate their widespread adoption in industrial use, and in pre-clinical trials^50^.

The landscape for new drug development is one of the most daunting of any product development pipeline. The estimated average cost for pharmaceutical companies to bring a new drug to market is approximately 2 billion dollars, and the process can take upwards of 15 years^33,51^. Despite this huge investment of time and money, the clinical success rate of drug approval stands between 10 and 20%^52,53^. This problem, however, is not new. The high attrition rate and overall decline in pharmaceutical research and development efficiency has been well documented for over 30 years^54,55^. A recurring culprit has been animal models, which are not sufficiently predictive of the safety nor efficacy of therapeutic compounds in humans^29,56,57^. The United States Food and Drug Administration (FDA) traditionally required data from animal studies, such as safe starting doses and potential toxicity levels, to ensure that human clinical trials can be safely conducted. While traditional high-throughput drug screening methods are effective at eliminating ineffective drugs, the process is often not conducive for these early stage systemic toxicity studies^58^. As an alternative model to animal studies, Organs on Chips provide this safety information as well as genetic toxicity, pharmacokinetics, ADME (absorption, distribution, metabolism, and excretion), reproductive toxicity, and carcinogenicity^34,59^. Aside from the direct effects of a potential drug on the tissue of interest, as would be screened for in high-throughput drug screenings, Organs on Chips have the ability to address systemic toxicity concerns via the linkage of several different types of Organ Chips^60^. Through the linkage of different chips, the effect of drugs on liver function or kidney function can be tested in conjunction with the effect of the drug on a primary tissue^3,4,61^. The use of multiple chips in series and parallel with each other can mimic the natural progression of a drug through different body compartments and provide more information on the potential systemic toxicity of a drug, aiding in the exclusion of toxic drugs at an earlier stage in the drug identification process^5,62^.

**Fig. 1:**
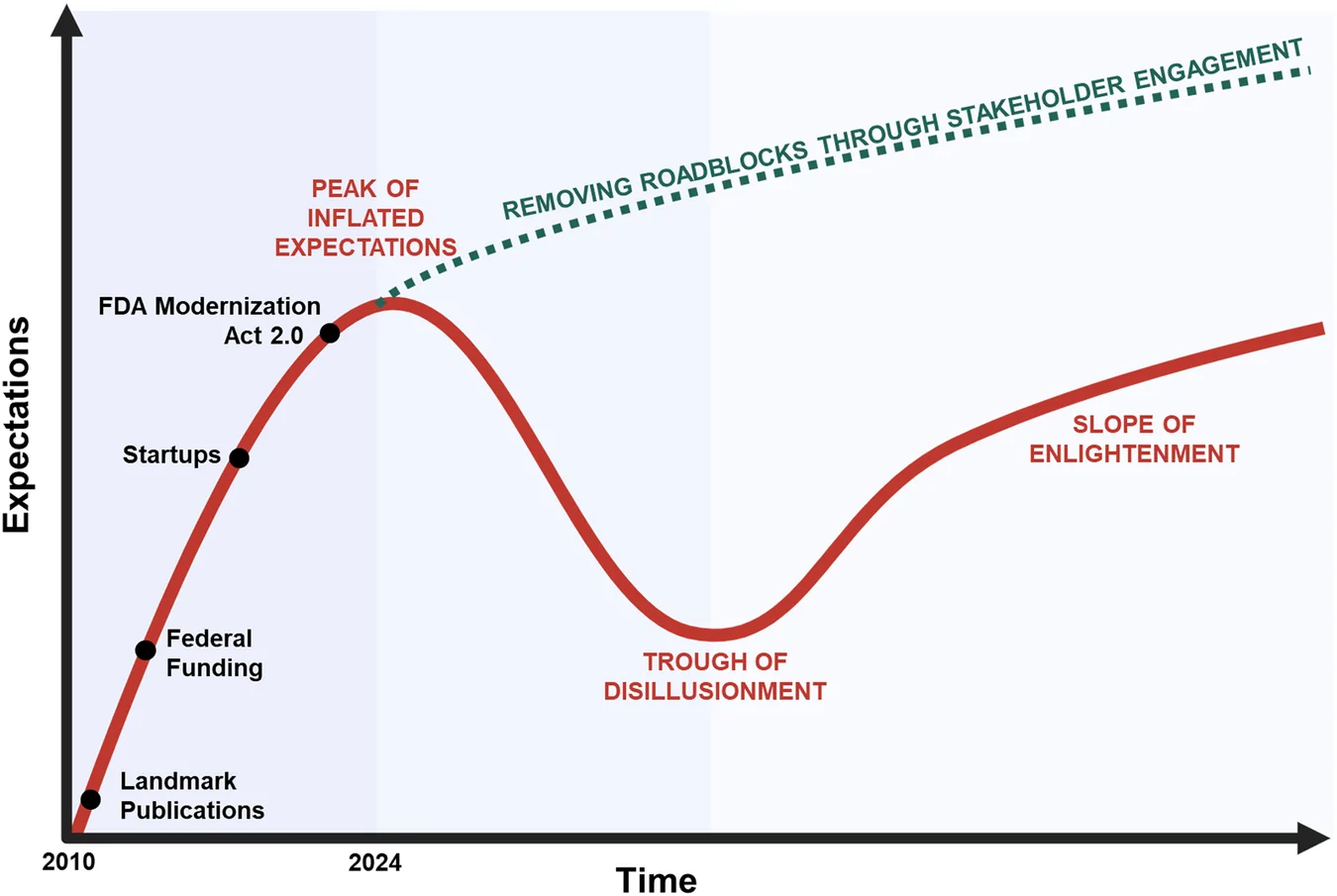
Gartner hype cycle for Organs on Chips. The Gartner hype cycle for Organs on Chips demonstrates how key events in the development of Organs on Chips can stimulate expectations of Organs on Chips within the scientific and general community. It also warns of the potential disillusionment that can occur if Organ on Chip dissemination is limited by a lack of stakeholder engagement.

The US Food and Drug Administration (FDA) and other regulatory agencies are conceptually supportive of new approaches and have communicated strategies that are being considered to put this acceptance into practice. To this end, the FDA formed an Alternative Methods Working Group focused on standardization of both new and existing in vitro models to ensure that the data that are generated are comparable and of the highest quality, and that studies are robust, reproducible, relevant, and fit for purpose. The Alternative Methods Group also facilitates interactions with global regulatory bodies interested in implementing alternative methods in toxicology. In response to the 21st Century Cures Act of 2016, FDA also launched its Innovative Science and Technology Approaches for New Drugs (ISTAND) Pilot Program, which is designed to support the development and, in some cases, qualification of novel approaches to drug development for use in regulatory decision making. Further, with the passage of the FDA Modernization Act 2.0 in 2023, animal testing requirements have changed and opened a pathway forward for widespread use of Organs on Chips. The excitement around the prospect of Organ on Chip technology to make a significant and sustained impact on drug discovery pipeline could not be higher. Applying the Gartner hype cycle paradigm for emerging technologies to Organs on Chips (Fig. 1), if the initial landmark publication and bold funding investments were the initial ‘innovation trigger’, we might be at the ‘peak of inflated expectations’ now. We submit that if technology providers, intended technology consumers, and regulatory agencies engage early and often, Organs on Chips can perhaps bypass the ‘trough of disillusionment’ that’s often triggered by implementation failures. Concerted efforts of all stakeholders can help define broad market applicability and relevance of these novel technologies, eventually leading to mainstream adoption.

## Organ on Chip stakeholders

**Fig. 2:**
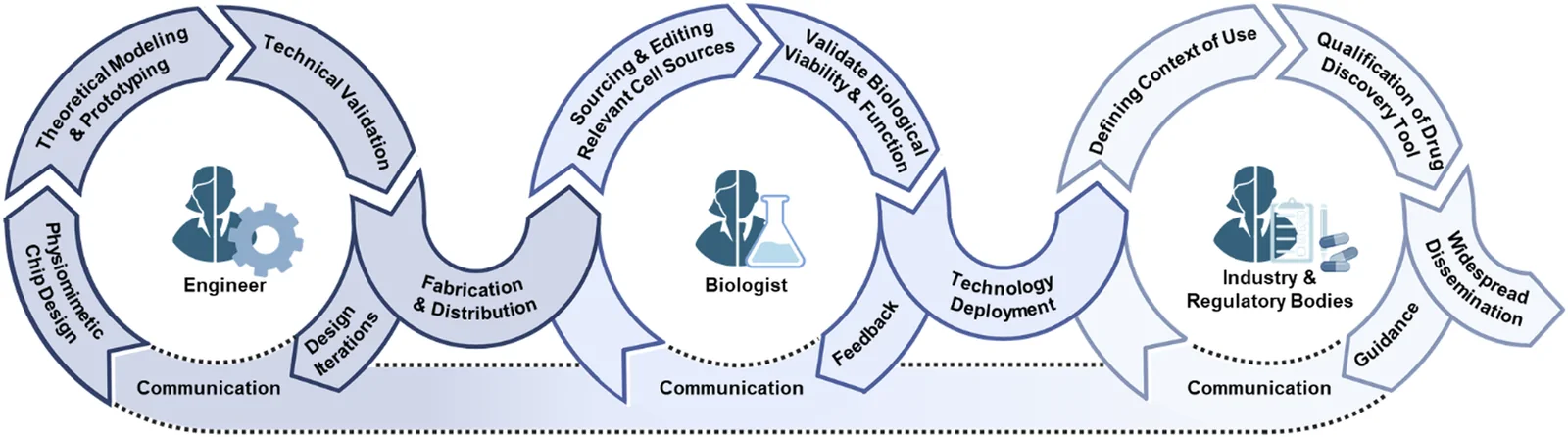
Stakeholder engagement in the iterative development process of Organs on Chips. A visual representation of the cyclic and iterative process that is critical to the development and usage of Organs on Chips. Within each segment, the general roles of individual stakeholders are highlighted in their individual iterative process as well as their role moving towards widespread dissemination of Organs on Chips.

Here we will discuss the roles and responsibilities of stakeholders within Organ on Chip development: engineering developers, biologist adopters, Organ on Chip startups, pharmaceutical industry, and regulatory bodies (illustrated in Fig. 2). It is important to note that while the roles and responsibilities of each of these parties differ, they are not wholly disjointed. It is also not an exhaustive list of all stakeholders. However, the design and dissemination of a validated Organ on Chip that is readily available, usable, and of value to end-users requires collaboration and partnership between all these stakeholders.

Engineers are responsible for bringing the engineering skills and knowledge necessary to construct a functional Organ on Chip and its associated control systems in an iterative design, build, test process. It is imperative for engineers to properly understand the in-vivo biomechanical and environmental niche for translation into a minimal functional unit that can represent tissue- and organ-level functions. Other biomechanical and environmental factors to consider may include culture temperature, flow directionality, basement membrane characteristics, biochemical gradients, oxygenation, and mechanical perturbations, etc. Not only should the biomedical engineer consider the physical and biological needs of a modeled system, but also the experimental desires of the end-user, whomever that may be. Chips should be designed to interface with established experimental tools and methods of analyses such as live-cell imaging, analyte collection, electrophysiological readouts, and any other offline tools. The materials used and mode of fluidic control should also be made with the end-users manufacturing capabilities, skillset, and desired scalability in mind.

Biologists are in part responsible for appropriately identifying the minimal functional unit necessary to address a specific biological question, its biological components, and investigative needs. In the instance of non-existent or poorly functioning cell lines, they help in the development of the cell lines, tissues, organoids and assembloids to be studied within Organs on Chips. As the stakeholder that brings the most biological knowledge to Organ on Chip development, the biologist is also responsible for helping to validate the biological and functional readouts from the device. As a cyclic development process, their role is neither truly at the end or beginning of the development and is essential at all stages.

The role of Organ on Chip startups and related industry is to establish the translational relevance of their technologies. They must understand the performance metrics, ideal context of use, and performance limitations of their platforms so that they can be benchmarked favorably against current standards, and so that they may appropriately interpret the results from pre-clinical trials. This is also central to being able to effectively manage investor and public expectations of device capabilities. Regulatory bodies assess the readiness of these approaches for specific context of uses, and ultimately make the decision to qualify them as a drug discovery tool.

As one of the primary end-users, the pharmaceutical industry evaluates the value proposition of a specific Organ on Chip technology based on whether it can be used for internal decision making, or if indeed, the technology has been qualified as a drug discovery tool. Important considerations such as throughput of readout, reproducibility of data, and compatibility with automation often drive decision making. Other potential end-users include disease biology laboratories that often do not have traditional engineering skills or microfluidics experience. Thus, user needs can vary greatly; hence the design and dissemination strategy of Organs on Chips requires careful consideration of the context within which platform will be ultimately used.

## Materials and manufacturing of Organs on Chips

**Fig. 3:**
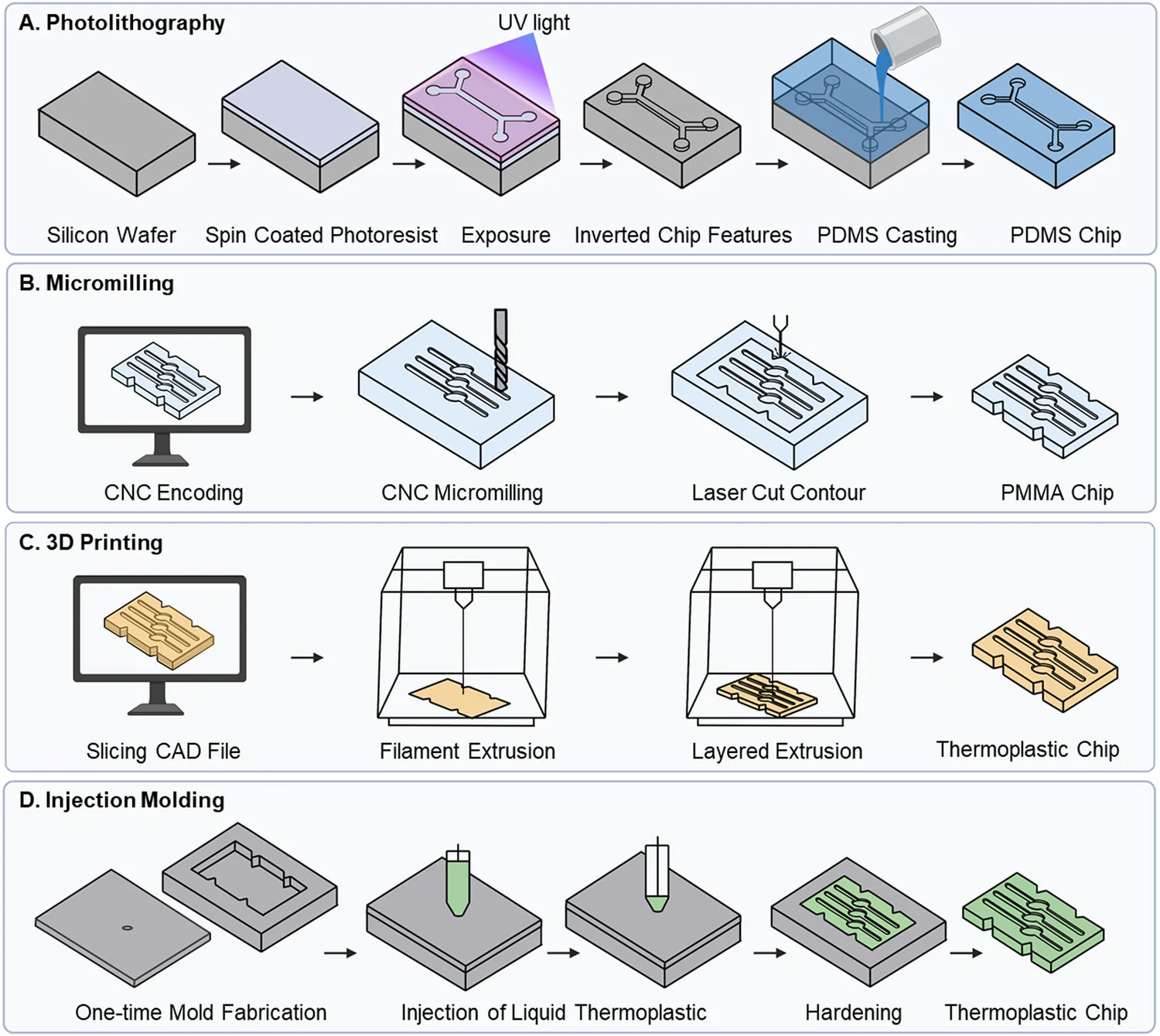
PDMS and plastic based manufacturing methods for Organs on Chips. **A** Photolithography based Organ on Chip fabrication begins with the formation of a mold with raised features via standard photolithography methods. PDMS can then be cast onto the mold to form a PDMS chip with negative spaces that form the microfluidic channels of the final PDMS-based Organ on Chip. **B** Micromilled Organs on Chip devices are milled via subtractive manufacturing techniques. Micromachining tools remove excess material from a plastic substrate resulting in the formation of the channels and features of an individual Organ on Chip. **C** 3D printed Organs on Chips are formed by the sequential deposition of a thermoplastic in thin layers to form the shape and features of an Organ on Chip. **D** Injection molded Organs on Chips start by the creation of a reusable external mold with positive features that correspond to negative spaces in the Organ on Chip design. The mold can then be filled with a liquid thermoplastic. Once the thermoplastic has set, the molded Organ on Chip can be removed from the mold.

Traditional polydimethylsiloxane (PDMS)-based microfluidic devices utilize a combination of photolithography and soft lithography to generate enclosed microscale channels for fluidics and cell culture (Fig. 3A). Just over a decade after its discovery, this method was adapted to layer intricate microfluidic channel geometries and porous structures to develop the first Organ on Chip, and the majority of subsequent Organ on Chip designs^5,12,63^. PDMS has become the material of choice for Organs on Chips due to properties that make it suitable for biological applications, such as low cytotoxicity, optical transparency, gas permeability, and established fabrication methods^64,65^. Despite the adoption of PDMS as the standard material for prototyping Organs on Chips, many limitations have been identified^5,66^. Adsorption of hydrophobic compounds and leaching of un-crosslinked oligomers have provided the greatest hindrance to the adoption of PDMS-based Organs on Chips for drug discovery assays. Significant work has been done to model the adsorptive properties of PDMS to optimize design parameters that minimize adsorption and mitigate the impact of PDMS on drug discovery studies^67,68^. Aside from optimizing Organ on Chip geometries, PDMS adsorption has been combated by the use of coatings such as parylene c and polyphenol that decrease gas and solvent permeability in PDMS^68,69^. Additionally, due to the limitations of the photolithographic fabrication method, the channels in PDMS-based microfluidic devices have a maximum height of approximately 200μm^70^, which typically prohibits their use for culture of large spheroids and organoids. Given the inherent limitations of PDMS, there is a need to develop Organs on Chips from alternative materials.

Within the Organ on Chip community, the problems with PDMS are well known. There is extensive literature on the fabrication and use of plastic based microfluidic devices, which may serve as an alternative to PDMS based Organs on Chips^71–74^. Plastics such as polymethyl methacrylate (PMMA or acrylic) became a popular alternative to PDMS because of their manufacturing ease and similar biological and experimental benefits, such as optical clarity and being bioinert^3,4,61,72,75^. Plastic-based Organs on Chips can be fabricated by methods such as micromilling, 3D printing, and injection molding, that each offer a benefit at different stages of development and use^74,76^.

Thermoplastic-based Organs on Chips can readily be fabricated via micromilling (Fig. 3B), a subtractive manufacturing technique where selective removal of a base substrate generates microscale features in the substrate^3,4,61,77^. Organs on Chips with a similar level of complexity to traditional PDMS-based Organs on Chips can be fabricated by micromilling machines using computer numerical control. Computer aided design models are implemented using software such as AutoCAD or SolidWorks. Once translated into milling code, a multi-axis mill removes material from the workpiece to form the final chip design. The size limitations of micromilled features depend on the minimum step size of the axis motors and the total number of axes; more advanced milling machines often have smaller motor steps and more moveable axes that allow for fabrication of smaller and more complex features^3,61,78,79^. Micromachined platforms are also more accessible for in-house prototyping as micromilling machine can easily be setup in most lab spaces without a cleanroom^78,79^. In-house prototyping also allows for rapid iteration between new designs.

As with micromilling, 3D printing can be utilized to fabricate complex Organs on Chips. 3D printing is a form of additive manufacturing that relies on the sequential deposition of substrate layers to form a final product (Fig. 3C). While there are several types of 3D printers, including polyjet printing, selective laser sintering, and stereolithography, they all rely on the localized deposition or solidification of material in a single XY plane. Once the individual layer is complete, the extrusion head or platform moves in the Z axis to start fabrication on a new layer. The overall complexity is limited by the minimal layer height and XY resolution of the printer used. By sequential layering material, 3D printing allows for the fabrication of complex overhangs and structures that are impossible to form by micromilling or injection molding. As with micromilling, 3D printers can be a valuable tool for in-house rapid prototyping due in part to the low cost and small lab footprint required to set up a 3D printer. With the use of larger and more complex 3D printers, the fabrication of Organs on Chips by 3D printing can be scaled to industrial use. As such, 3D printing is useful for the development phase of Organs on Chips as well as the production for widescale use.

Once an Organ on Chip design is finalized, a popular method for its mass production is injection molding (Fig. 3D). This process is very cost-efficient at large scales and can rapidly produce identical plastic-based devices. The process requires that a negative mold be machined, in two or more parts that can be clamped together to form a mold with a cavity that mimics the designed final product. Thermoplastics are then heated up and injected into the mold completely filling the cavity. The plastic is then allowed to cool and set within the mold before being taken out. This process can be easily scaled by serially filling sets of linked base molds at a time and removing the excess material between plastic devices post-fabrication. Because the molds can be reused numerous times, the process has a high start-up cost, but is incredibly cost-efficient when used at a large scale. The cost-efficiency of injection molding makes it a prime fabrication technique for industrial use of Organs on Chips while its high start-up costs may limit its use for initial prototyping.

Plastic microfluidic devices, fabricated through micromilling, 3D printing, and injection molding are a viable alternative to PDMS for Organs on Chips. Each of these methods comes with benefits and drawbacks that may alter which method is used depending on the development stage of an Organ on Chip or the end-user. Regardless of the manufacturing method, plastic-based Organs on Chips are becoming more popular at all stages of Organ on Chip development. It is especially important that they be considered early in the prototyping stage to avoid the need to revalidate Organ on Chip devices as they move between stakeholders and require iterative redesigning. However, widespread dissemination and use of Organs on Chips also requires the development of a complete microfluidic system, including peripheral support and analytic devices that allow Organs on Chips to be usable to perform a wide array of biologic experiments.

## Platform development for Organ on Chip operation

From a broader perspective, a complete Organ on Chip system requires additional components, such as electronics, software and fluid handling systems. Therefore, a systems engineering approach is necessary to design and manage the various components required for a practical platform^80–82^. This often requires a multidisciplinary engineering approach that combines mechanical, electrical, and computer engineering, with the added unique considerations of developing living biologic systems^83^. Based on the end-user needs and workflow, basic functional elements are conceptualized and arranged to develop the platform configuration. Next, functional elements are identified and grouped to determine the most vital components of the platform. Organization elements at this stage can help identify key features of the design and decrease time required to address potential failure modes^84^. This process can also be used to effectively bring stakeholders together and facilitate conversation around needs and expectations. Overall, careful planning and understanding of the processes involved in operation of an Organs on Chips is vital for the development of useful devices and systems. In the coming adolescence of Organs on Chips, we are starting to see the development of more complete platforms, which are only just becoming commercially available.

## Pathway to adoption

Translational relevance is the anchor for any new non-clinical model system (cell based assay, Organ on Chip platform, or animal model) that is intended to help understand, prevent, or treat a human condition. The principles that define translational relevance include: replication of human physiology and pathophysiology (organ responses or disease state/pathogenesis), genetics, epigenetics and/or the molecular biology of human cells. New approach methodologies such as Organs on Chips will be expected to meet translational expectations if they are to be broadly adopted to decrease reliance on animal models in drug discovery and biomedical research. Gaining sufficient legitimacy based on the performance (track record) of the approach is key to the successful adoption by scientists and organizations. Once a method demonstrates that it is equivalent or superior to other models for predicting or explaining human conditions or outcomes, it may be adopted as a model of choice. Ultimately, an Organ on Chip’s performance determines whether the technology or approach can be used to answer a defined and specific research question. Although all new approaches start with performance uncertainty, resource investments that serve to reduce the uncertainty are required to accelerate adoption. Important components of a pathway to adoption, and their inclusion in a drug discovery pipeline include Validation, defining Context of Use, Qualification as a drug discovery tool that is Fit for Purpose, and finally, Benchmarking^85,86^.

Validation process starts with assessments of platform sensitivity, specificity, precision, robustness, reproducibility, and stability. It also seeks to define the similarity between the endpoint generated by the Organ on Chip and the clinical readouts, as well as the predictive demonstration of clinically effective interventions producing a similar effect in an Organ on Chip platform^87^. Defining Context of Use involves identifying the exact utility of the emerging technology. It describes the conditions under which the Organ on Chip is to be used successfully in performance of a task, and what one should expect it to do. Qualification is the process of determining whether the Organ on Chip is suitable for its intended purpose based on pre-determined performance criteria. Qualification process is also intended to explain how results of the approach will be used in decision-making^88^. A Fit for Purpose statement indicates that the Organ on Chip assay or approach has “good enough” validation/qualification information to support its context of use^89,90^. Ultimately, the end-user will often conduct Benchmarking studies to rigorously compare the performance of different methods using well-characterized internal benchmark datasets, to provide guidance for improvements in the current platform, or guide investment in alternative platforms.

Qualification scheme for any emerging technology or method is highly dependent upon the complexity of the questions (or mechanistic hypotheses) and the pathophysiology necessary to answer these questions^91,92^. For example, if an Organ on Chip technology is expected to reduce reliance on an animal model of pulmonary fibrosis, then the new approach would be expected to recapitulate the underlying molecular and cellular mechanisms that are present and relevant in humans. Expectations for stringency of the qualification process relate to how the data will be used, the decisions they will inform, and the potential consequences of those decisions for human health. For example, the expectations for stringency may differ when the context of use is the safety evaluation of a novel drug versus a basic biology application such as understanding host-pathogen interactions^93^.

## Summary

The rapid development of Organs on Chips, primarily driven by interest from pharmaceutical companies, has led to a need for industry standards for the field. However, standardization cannot stifle innovation. Properly addressing the translational barriers for various applications is key for Organs on Chips to reach their ultimate potential. While the resolution of these barriers should diminish the trepidation of slow adopters, dialog with industry partners will help in defining important milestones, such as throughput, cost per data point, and demonstration of value proposition in terms of cost and time savings. By facilitating an understanding of individual stakeholder roles in the development of Organs on Chips, they can be more easily implemented at multiple stages of drug discovery and development. However, deliberate care should be taken to avoid narrowing the scope of Organ on Chip technology solely to drug discovery and high-throughput applications desired by pharmaceutical companies. Instead, all the potential end-users should be kept in mind throughout the development and dissemination phases of Organ on Chip technologies. The widespread dissemination of Organs on Chips requires a recognition that this highly interdisciplinary effort is not only scientifically fertile, but also requires ushering in a new framework for scientific endeavors where engineers, scientists, industry, funding agencies, investors, and regulatory bodies are all contributing at each phase of development for these highly innovative and impactful systems.

## Outlook

Once the framework for the widespread dissemination and deployment of Organs on Chips herein proposed is accepted and used by the general scientific community, Organs on Chips have the potential to become a core research tool for basic biological sciences, translational clinical sciences, and also to aid the passage of novel therapeutics from bench to bedside. For basic biological science research, Organs on Chips can provide models of many organ systems—either through the culture of ex-vivo tissue samples or tissue engineered samples—that match the cellular, physiologic, and genetic profile of a human more accurately than 2D culture models or animal models. Translational sciences like the pharmaceutical industry will greatly benefit from the high throughput testing of potential therapeutics within Organs on Chips without risking the human insensitivities that have plagued traditional animal models in the pre-clinical testing phase, such as the loss of targeting when moving from mouse to human models or the presence of toxicities in humans that are not present in other species. Looking beyond the uses of Organs on Chips in the realms of research and development, their widespread usage in basic sciences and industry as well as their continued adoption by regulatory bodies will help open up the potential clinical uses of Organs on Chips for personalized medicine. Through the use of cell lines derived from an individual patient, Organs on Chips could be used to identify therapeutics that have the most specific impact for the patient while also minimizing their individual risk for adverse side effects. Organs on Chips have the potential to be widely used in a clinical setting to identify the best option from a variety of drug options just as bacterial cultures are used to select for specific antibiotics. In order to unlock these practical and promising clinical uses of Organs on Chips, we must begin by encouraging the widespread dissemination and deployment of Organs on Chips within the scientific community and relevant regulatory bodies. Without further dissemination, Organs on Chips are at risk of becoming a niche experimental tool. While trying to position an Organ on Chip platform within mainstream drug discovery, it is reasonable to adopt principles of translational relevance to define qualification and/or validation pathways and to establish a well-defined context of use. Following the framework we have herein described, the scientific community and regulatory bodies can collaborate to form best practices for the use of Organs on Chips and facilitate their adoption within new areas of research. As Organs on Chips become more widely used and accessible, the full potential of these devices may be realized.

## References

[1] Bhatia, S. N. & Ingber, D. E. Microfluidic organs-on-chips. *Nat. Biotechnol.* **32**, 760–772 (2014). 10.1038/nbt.2989 25093883

[2] Leung, C. M. et al. A guide to the organ-on-a-chip. *Nat. Rev. Methods Prim.* **2**, 33 (2022).

[3] Alver, C. G. et al. SliceChip: a benchtop fluidic platform for organotypic culture and serial assessment of human and rodent pancreatic slices. *Lab. Chip* **24**, 1557–1572 (2024). 10.1039/d3lc00850a PMC10939771 38205530

[4] McDuffie, D. et al. Acrylic-based culture plate format perfusion device to establish liver endothelial–epithelial interface. *Lab. Chip* **23**, 3106–3119 (2023). 10.1039/d3lc00382e PMC10351567 37313651

[5] Ronaldson-Bouchard, K. et al. A multi-organ chip with matured tissue niches linked by vascular flow. *Nat. Biomed. Eng.* **6**, 351–371 (2022). **Modules of phenotypically mature human heart, liver, bone and skin tissue niches were modularly connected through vascular channel for up to 4 weeks**. 10.1038/s41551-022-00882-6 PMC9250010 35478225

[6] Vunjak-Novakovic, G., Ronaldson-Bouchard, K. & Radisic, M. Organs-on-a-chip models for biological research. *Cell* **184**, 4597–4611 (2021). 10.1016/j.cell.2021.08.005 PMC8417425 34478657

[7] Zhao, Y. et al. A Platform for Generation of Chamber-Specific Cardiac Tissues and Disease Modeling. *Cell* **176**, 913–927.e918 (2019). 10.1016/j.cell.2018.11.042 PMC6456036 30686581

[8] Bowles, A. C., Ishahak, M. M., Glover, S. J., Correa, D. & Agarwal, A. Evaluating Vascularization of Heterotopic Islet Constructs for Type 1 Diabetes Using an In Vitro Platform. *Integr. Biol.* **11**, 331–341 (2019). 10.1093/intbio/zyz027 31724717

[9] Besser, R. R. et al. Enzymatically crosslinked gelatin-laminin hydrogels for applications in neuromuscular tissue engineering. *Biomater. Sci.* **8**, 591–606 (2020). 10.1039/c9bm01430f PMC7141910 31859298

[10] Besser, R. R. et al. A Chemically Defined Common Medium for Culture of C2C12 Skeletal Muscle and Human Induced Pluripotent Stem Cell Derived Spinal Spheroids. *Cell. Mol. Bioeng.* **13**, 605–619 (2020). 10.1007/s12195-020-00624-1 PMC7704992 33281990

[11] Ajay, A. K. Functional Drug Screening using Kidney Cells On-A-Chip: Advances in Disease Modeling and Development of Biomarkers. *Kidney360* **3**, 194–198 (2022). 10.34067/KID.0007172021 PMC8967633 35373124

[12] Huh, D. et al. Reconstituting organ-level lung functions on a chip. *Science* **328**, 1662–1668 (2010). **Seminal lung on a chip demonstration that kickstarted the Organ on Chip momentum from chip developers, startup companies, and funding and regulatory agencies**. 10.1126/science.1188302 PMC8335790 20576885

[13] Dasgupta, Q. et al. A human lung alveolus-on-a-chip model of acute radiation-induced lung injury. *Nat. Commun.* **14**, 6506 (2023). 10.1038/s41467-023-42171-z PMC10579267 37845224

[14] Bai, H. et al. Mechanical control of innate immune responses against viral infection revealed in a human lung alveolus chip. *Nat. Commun.* **13**, 1928 (2022). 10.1038/s41467-022-29562-4 PMC8993817 35396513

[15] Musah, S. et al. Mature induced-pluripotent-stem-cell-derived human podocytes reconstitute kidney glomerular-capillary-wall function on a chip. *Nat. Biomed. Eng.* **1**, 0069 (2017). 10.1038/s41551-017-0069 PMC5639718 29038743

[16] Picollet-D’hahan, N., Zuchowska, A., Lemeunier, I. & Le Gac, S. Multiorgan-on-a-Chip: A Systemic Approach To Model and Decipher Inter-Organ Communication. *Trends Biotechnol.* **39**, 788–810 (2021). 10.1016/j.tibtech.2020.11.014 33541718

[17] Zhang, Y. S. et al. Multisensor-integrated organs-on-chips platform for automated and continual in situ monitoring of organoid behaviors. *Proc. Natl Acad. Sci.* **114**, E2293–E2302 (2017). 10.1073/pnas.1612906114 PMC5373350 28265064

[18] Tao, T. et al. Microengineered Multi-Organoid System from hiPSCs to Recapitulate Human Liver-Islet Axis in Normal and Type 2 Diabetes. *Adv. Sci.* **9**, 2103495 (2022). 10.1002/advs.202103495 PMC8844474 34951149

[19] Wagner, I. et al. A dynamic multi-organ-chip for long-term cultivation and substance testing proven by 3D human liver and skin tissue co-culture. *Lab Chip* **13**, 3538–3547 (2013). 10.1039/c3lc50234a 23648632

[20] Oleaga, C. et al. Long-Term Electrical and Mechanical Function Monitoring of a Human-on-a-Chip System. *Adv. Funct. Mater.* **29**, 1805792 (2019). 10.1002/adfm.201805792 PMC9113405 35586798

[21] Shroff, T. et al. Studying metabolism with multi-organ chips: new tools for disease modelling, pharmacokinetics and pharmacodynamics. *Open Biol.* **12**, 210333 (2022). 10.1098/rsob.210333 PMC8889168 35232251

[22] Sun, X.-Y. et al. Generation of vascularized brain organoids to study neurovascular interactions. *eLife* **11**, e76707 (2022). 10.7554/eLife.76707 PMC9246368 35506651

[23] Paşca, S. P. Assembling human brain organoids. *Science* **363**, 126–127 (2019). 10.1126/science.aau5729 30630918

[24] Kanton, S. & Paşca, S. P. Human assembloids. *Development* **149**, dev201120 (2022). 10.1242/dev.201120 36317797

[25] Xiao, S. et al. A microfluidic culture model of the human reproductive tract and 28-day menstrual cycle. *Nat. Commun.* **8**, 14584 (2017). **A remarkable study that simulates the** in vivo **female reproductive tract by functional connection of endocrine loops between the ovary, fallopian tube, uterus, cervix and liver modules**. 10.1038/ncomms14584 PMC5379057 28350383

[26] Young, R. E. & Huh, D. D. Organ-on-a-chip technology for the study of the female reproductive system. *Adv. Drug Deliv. Rev.* **173**, 461–478 (2021). 10.1016/j.addr.2021.03.010 PMC8178221 33831478

[27] Mahajan, G. et al. Vaginal microbiome-host interactions modeled in a human vagina-on-a-chip. *Microbiome* **10**, 201 (2022). 10.1186/s40168-022-01400-1 PMC9701078 36434666

[28] Patel, S. N. et al. Organoid microphysiological system preserves pancreatic islet function within 3D matrix. *Sci. Adv.* **7**, eaba5515 (2021). 10.1126/sciadv.aba5515 PMC7880596 33579705

[29] Ingber, D. E. Human organs-on-chips for disease modelling, drug development and personalized medicine. *Nat. Rev. Genet*. **23**, 467–491 (2022). 10.1038/s41576-022-00466-9 PMC8951665 35338360

[30] Low, L. A. & Tagle, D. A. Organs-on-chips: Progress, challenges, and future directions. *Exp. Biol. Med.* **242**, 1573–1578 (2017). 10.1177/1535370217700523 PMC5661765 28343437

[31] Alassaf, A. et al. Engineering anisotropic cardiac monolayers on microelectrode arrays for non-invasive analyses of electrophysiological properties. *Analyst* **145**, 139–149 (2020). 10.1039/c9an01339c 31746833

[32] Alassaf, A., Ishahak, M., Bowles, A. & Agarwal, A. Microelectrode Array based Functional Testing of Pancreatic Islet Cells. *Micromachines* **11**, 507 (2020). 10.3390/mi11050507 PMC7281363 32429597

[33] Ma, C., Peng, Y., Li, H. & Chen, W. Organ-on-a-Chip: A New Paradigm for Drug Development. *Trends Pharm. Sci.* **42**, 119–133 (2021). 10.1016/j.tips.2020.11.009 PMC7990030 33341248

[34] Ewart, L. et al. Performance assessment and economic analysis of a human Liver-Chip for predictive toxicology. *Commun. Med.* **2**, 154 (2022). 10.1038/s43856-022-00209-1 PMC9727064 36473994

[35] Kroll, K. T. et al. Immune-infiltrated kidney organoid-on-chip model for assessing T cell bispecific antibodies. *Proc. Natl Acad. Sci.* **120**, e2305322120, 10.1073/pnas.2305322120 (2023). PMC10467620 37603766

[36] Jang, K.-J. et al. Reproducing human and cross-species drug toxicities using a Liver-Chip. *Sci. Transl. Med.* **11**, eaax5516 (2019). **Liver-Chip consisting of rat, dog, or human hepatocytes, endothelial cells, Kupffer cells, and stellate cells detected diverse phenotypes of liver toxicity, and species-specific toxicities**. 10.1126/scitranslmed.aax5516 31694927

[37] Esch, E. W., Bahinski, A. & Huh, D. Organs-on-chips at the frontiers of drug discovery. *Nat. Rev. Drug Discov.* **14**, 248–260 (2015). 10.1038/nrd4539 PMC4826389 25792263

[38] Atkins, J. T. et al. Pre-clinical animal models are poor predictors of human toxicities in phase 1 oncology clinical trials. *Br. J. Cancer* **123**, 1496–1501 (2020). 10.1038/s41416-020-01033-x PMC7652903 32868897

[39] Marshall, L. J., Bailey, J., Cassotta, M., Herrmann, K. & Pistollato, F. Poor Translatability of Biomedical Research Using Animals — A Narrative Review. *Alternatives Lab. Anim.* **51**, 102–135 (2023). 10.1177/02611929231157756 36883244

[40] Pol, S. U. et al. Network-Based Genomic Analysis of Human Oligodendrocyte Progenitor Differentiation. *Stem Cell Rep.* **9**, 710–723 (2017). 10.1016/j.stemcr.2017.07.007 PMC5550273 28793249

[41] Peel, S. et al. Introducing an automated high content confocal imaging approach for Organs-on-Chips. *Lab Chip* **19**, 410–421 (2019). 10.1039/c8lc00829a 30663729

[42] Azizgolshani, H. et al. High-throughput organ-on-chip platform with integrated programmable fluid flow and real-time sensing for complex tissue models in drug development workflows. *Lab Chip* **21**, 1454–1474 (2021). **96 Organ on Chips representing liver, vascular, gut, and kidney tissues were integrated on a single operating platform with electrical, optical readouts and high content screening capabilities**. 10.1039/d1lc00067e 33881130

[43] Fisher, C. R. et al. A High-Throughput, High-Containment Human Primary Epithelial Airway Organ-on-Chip Platform for SARS-CoV-2 Therapeutic Screening. *Cells* **12**, 2639 (2023). 10.3390/cells12222639 PMC10669988 37998374

[44] Palasantzas, V. E. J. M. et al. iPSC-derived organ-on-a-chip models for personalized human genetics and pharmacogenomics studies. *Trends Genet.* **39**, 268–284 (2023). 10.1016/j.tig.2023.01.002 36746737

[45] Zhao, Y., Wang, E. Y., Lai, F. B. L., Cheung, K. & Radisic, M. Organs-on-a-chip: a union of tissue engineering and microfabrication. *Trends Biotechnol.* **41**, 410–424 (2023). 10.1016/j.tibtech.2022.12.018 PMC9985977 36725464

[46] Singh, D., Mathur, A., Arora, S., Roy, S. & Mahindroo, N. Journey of organ on a chip technology and its role in future healthcare scenario. *Appl. Surface Sci. Adv.* **9**, 10.1016/j.apsadv.2022.100246 (2022).

[47] Zhang, B. & Radisic, M. Organ-on-a-chip devices advance to market. *Lab Chip* **17**, 2395–2420 (2017). **A comprehensive look at startup activity spurred on academic labortories that were early pioneers in singular and connected multiple Organ on Chip Platforms**. 10.1039/c6lc01554a 28617487

[48] da Silva, R. G. L. & Blasimme, A. Organ chip research in Europe: players, initiatives, and policies. *Front. Bioeng. Biotech.* **11**, 1237561 (2023). 10.3389/fbioe.2023.1237561 PMC10507620 37731764

[49] Franzen, N. et al. Impact of organ-on-a-chip technology on pharmaceutical R&D costs. *Drug Discov. Today* **24**, 1720–1724 (2019). 10.1016/j.drudis.2019.06.003 31185290

[50] Hargrove-Grimes, P., Low, L. A. & Tagle, D. A. Microphysiological Systems: Stakeholder Challenges to Adoption in Drug Development. *Cells Tissues Organs* **211**, 269–281 (2021). 10.1159/000517422 PMC8831652 34380142

[51] Simoens, S. & Huys, I. R&D Costs of New Medicines: A Landscape Analysis. *Front. Med.* **8**, 760762 (2021). 10.3389/fmed.2021.760762 PMC8576181 34765624

[52] Wong, C. H., Siah, K. W. & Lo, A. W. Estimation of clinical trial success rates and related parameters. *Biostatistics* **20**, 273–286 (2019). 10.1093/biostatistics/kxx069 PMC6409418 29394327

[53] Yamaguchi, S., Kaneko, M. & Narukawa, M. Approval success rates of drug candidates based on target, action, modality, application, and their combinations. *Clin. Transl. Sci.* **14**, 1113–1122 (2021). 10.1111/cts.12980 PMC8212735 33831276

[54] Scannell, J. W. et al. Predictive validity in drug discovery: what it is, why it matters and how to improve it. *Nat. Rev. Drug Discov.* **21**, 915–931 (2022). 10.1038/s41573-022-00552-x 36195754

[55] Schuhmacher, A. et al. Analysis of pharma R&D productivity – a new perspective needed. *Drug Discov. Today* **28**, 103726 (2023). 10.1016/j.drudis.2023.103726 37506762

[56] Collins, F. S. Reengineering translational science: the time is right. *Sci. Transl. Med.* **3**, 90cm17 (2011). 10.1126/scitranslmed.3002747 PMC5101940 21734173

[57] Adhikary, P. P., Ul Ain, Q., Hocke, A. C. & Hedtrich, S. COVID-19 highlights the model dilemma in biomedical research. *Nat. Rev. Mater.* **6**, 374–376 (2021). 10.1038/s41578-021-00305-z PMC7967778 33747552

[58] Blay, V., Tolani, B., Ho, S. P. & Arkin, M. R. High-Throughput Screening: today’s biochemical and cell-based approaches. *Drug Discov. Today* **25**, 1807–1821 (2020). 10.1016/j.drudis.2020.07.024 32801051

[59] Keuper-Navis, M. et al. The application of organ-on-chip models for the prediction of human pharmacokinetic profiles during drug development. *Pharmacol. Res.* **195**, 106853 (2023). 10.1016/j.phrs.2023.106853 37473876

[60] Koning, J. J. et al. A Multi-Organ-on-Chip Approach to Investigate How Oral Exposure to Metals Can Cause Systemic Toxicity Leading to Langerhans Cell Activation in Skin. *Front. Toxicol.* **3**, 824825 (2021). 10.3389/ftox.2021.824825 PMC8915798 35295125

[61] Ishahak, M. et al. Modular Microphysiological System for Modeling of Biologic Barrier Function. *Front. Bioeng. Biotech.* **8**, 581163 (2020). 10.3389/fbioe.2020.581163 PMC7693638 33304889

[62] Cong, Y. et al. Drug Toxicity Evaluation Based on Organ-on-a-chip Technology: A Review. *Micromachines* **11**, 381 (2020). 10.3390/mi11040381 PMC7230535 32260191

[63] Zamprogno, P. et al. Second-generation lung-on-a-chip with an array of stretchable alveoli made with a biological membrane. *Commun. Biol.* **4**, 168 (2021). 10.1038/s42003-021-01695-0 PMC7864995 33547387

[64] Sia, S. K. & Whitesides, G. M. Microfluidic devices fabricated in poly(dimethylsiloxane) for biological studies. *Electrophoresis* **24**, 3563–3576 (2003). 10.1002/elps.200305584 14613181

[65] Rein, C., Toner, M. & Sevenler, D. Rapid prototyping for high-pressure microfluidics. *Sci. Rep.* **13**, 1232 (2023). 10.1038/s41598-023-28495-2 PMC9868119 36683072

[66] van Meer, B. J. et al. Small molecule absorption by PDMS in the context of drug response bioassays. *Biochem. Biophys. Res. Commun.* **482**, 323–328 (2017). 10.1016/j.bbrc.2016.11.062 PMC5240851 27856254

[67] Shirure, V. S. & George, S. C. Design considerations to minimize the impact of drug absorption in polymer-based organ-on-a-chip platforms. *Lab. Chip* **17**, 681–690 (2017). 10.1039/c6lc01401a PMC10597325 28102869

[68] Reese, W. M. et al. Facile Macrocyclic Polyphenol Barrier Coatings for PDMS Microfluidic Devices. *Adv. Funct. Mater.* **30**, 2001274 (2020).

[69] Flueckiger, J., Bazargan, V., Stoeber, B. & Cheung, K. C. Characterization of postfabricated parylene C coatings inside PDMS microdevices. *Sens. Actuators B Chem.* **160**, 864–874 (2011).

[70] Jung, D. J. et al. A one-stop microfluidic-based lung cancer organoid culture platform for testing drug sensitivity. *Lab. Chip* **19**, 2854–2865 (2019). 10.1039/c9lc00496c 31367720

[71] Becker, H. & Locascio, L. E. Polymer microfluidic devices. *Talanta* **56**, 267–287 (2002). 10.1016/s0039-9140(01)00594-x 18968500

[72] Ren, K., Zhou, J. & Wu, H. Materials for microfluidic chip fabrication. *Acc. Chem. Res.* **46**, 2396–2406 (2013). 10.1021/ar300314s 24245999

[73] Gonçalves, I. M. et al. Recent trends of biomaterials and biosensors for organ-on-chip platforms. *Bioprinting* **26**, e00202 (2022).

[74] Schneider, S., Gruner, D., Richter, A. & Loskill, P. Membrane integration into PDMS-free microfluidic platforms for organ-on-chip and analytical chemistry applications. *Lab. Chip* **21**, 1866–1885 (2021). 10.1039/d1lc00188d 33949565

[75] Schneider, S. et al. Peristaltic on-chip pump for tunable media circulation and whole blood perfusion in PDMS-free organ-on-chip and Organ-Disc systems. *Lab. Chip* **21**, 3963–3978 (2021). 10.1039/d1lc00494h 34636813

[76] Radisic, M. & Loskill, P. Beyond PDMS and Membranes: New Materials for Organ-on-a-Chip Devices. *ACS Biomater. Sci. Eng.* **7**, 2861–2863 (2021). 10.1021/acsbiomaterials.1c00831 34275298

[77] Lenguito, G. et al. Resealable, optically accessible, PDMS-free fluidic platform for ex vivo interrogation of pancreatic islets. *Lab. Chip* **17**, 772–781 (2017). 10.1039/c6lc01504b PMC5330806 28157238

[78] Guckenberger, D. J., de Groot, T. E., Wan, A. M., Beebe, D. J. & Young, E. W. Micromilling: a method for ultra-rapid prototyping of plastic microfluidic devices. *Lab. Chip* **15**, 2364–2378 (2015). 10.1039/c5lc00234f PMC4439323 25906246

[79] Leclerc, C. A. et al. Rapid design and prototyping of microfluidic chips via computer numerical control micromilling and anisotropic shrinking of stressed polystyrene sheets. *Microfluidics Nanofluidics* **25**, 12 (2021).

[80] Low, L. A., Mummery, C., Berridge, B. R., Austin, C. P. & Tagle, D. A. Organs-on-chips: into the next decade. *Nat. Rev. Drug Discov.* **20**, 345–361 (2021). 10.1038/s41573-020-0079-3 32913334

[81] Sood, A., Kumar, A., Gupta, V. K., Kim, C. M. & Han, S. S. Translational Nanomedicines Across Human Reproductive Organs Modeling on Microfluidic Chips: State-of-the-Art and Future Prospects. *ACS Biomater. Sci. Eng.* **9**, 62–84 (2023). 10.1021/acsbiomaterials.2c01080 36541361

[82] Fuchs, S. et al. In-Line Analysis of Organ-on-Chip Systems with Sensors: Integration, Fabrication, Challenges, and Potential. *ACS Biomater. Sci. Eng.* **7**, 2926–2948 (2021). 10.1021/acsbiomaterials.0c01110 PMC8278381 34133114

[83] Rogal, J., Schlünder, K. & Loskill, P. Developer’s Guide to an Organ-on-Chip Model. *ACS Biomater. Sci. Eng.* **8**, 4643–4647 (2022). 10.1021/acsbiomaterials.1c01536 PMC9667877 35760397

[84] Ishahak, M. et al. Integrated platform for operating and interrogating organs-on-chips. *Anal. Methods* **11**, 5645–5651 (2019).

[85] Tadenev, A. L. D. & Burgess, R. W. Model validity for preclinical studies in precision medicine: precisely how precise do we need to be. *Mamm. Genome* **30**, 111–122 (2019). 10.1007/s00335-019-09798-0 PMC6606658 30953144

[86] Steger-Hartmann, T. & Raschke, M. Translating in vitro to in vivo and animal to human. *Curr. Opin. Toxicol.* **23-24**, 6–10 (2020).

[87] Patterson, E. A., Whelan, M. P. & Worth, A. P. The role of validation in establishing the scientific credibility of predictive toxicology approaches intended for regulatory application. *Comput. Toxicol.* **17**, 100144 (2021). 10.1016/j.comtox.2020.100144 PMC7903516 33681540

[88] Piergiovanni, M., Mennecozzi, M., Sampani, S. & Whelan, M. Heads on! Designing a qualification framework for organ-on-chip. *ALTEX Alternatives Anim. Exp.* **41**, 320–323 (2024). 10.14573/altex.2401231 38414285

[89] Parish, S. T. et al. An evaluation framework for new approach methodologies (NAMs) for human health safety assessment. *Regul. Toxicol. Pharm.* **112**, 104592 (2020). 10.1016/j.yrtph.2020.104592 32017962

[90] Pamies, D. et al. Recommendations on fit-for-purpose criteria to establish quality management for microphysiological systems and for monitoring their reproducibility. *Stem Cell Rep.* **19**, 604–617 (2024). 10.1016/j.stemcr.2024.03.009 PMC11103889 38670111

[91] Baran, S. W. et al. Perspectives on the evaluation and adoption of complex in vitro models in drug development: Workshop with the FDA and the pharmaceutical industry (IQ MPS Affiliate). *ALTEX Alternatives Animal Exp.* **39**, 297–314 (2022). **An important report from pharmaceutical industry partners and the FDA on the need to define context of use, and the qualification/validation pathway before widespread adoption of Organs on Chips**. 10.14573/altex.2112203 35064273

[92] Pognan, F. et al. The evolving role of investigative toxicology in the pharmaceutical industry. *Nat. Rev. Drug Discov.* **22**, 317–335 (2023). 10.1038/s41573-022-00633-x PMC9924869 36781957

[93] Alonso-Roman, R. et al. Organ-on-chip models for infectious disease research. *Nat. Microbiol.* **9**, 891–904 (2024). 10.1038/s41564-024-01645-6 38528150

